# Transmitter-assisted joint data-aided channel estimation and PAPR reduction scheme in wireless fading channels

**DOI:** 10.1038/s41598-025-33617-z

**Published:** 2026-02-10

**Authors:** Inaamullah Khan, Mohammad Mahmudul Hasan, Michael Cheffena

**Affiliations:** Faculty of Engineering, Norwegian University of Science and Technology (NTNU), 2815 Gjøvik, Norway

**Keywords:** Transmitter-assisted DACE, MIMO-OFDM, PAPR reduction, LS, LMMSE, Rayleigh fading channel, Rician fading channel, MSE, BER, Electrical and electronic engineering, Engineering

## Abstract

This paper presents a novel transmitter-assisted joint scheme that simultaneously addresses two critical challenges in modern wireless communication systems: peak-to-average power ratio (PAPR) reduction and accurate channel estimation. The proposed solution integrates modified gamma correction commanding (MGCC) with data-aided channel estimation (DACE) for both single-input single-output (SISO) and multiple-input multiple-output (MIMO) orthogonal frequency-division multiplexing (OFDM) wireless systems. The key novelty lies in the unique dual-functionality approach, where high-peak power carriers, traditionally a source of distortion due to high PAPR, are repurposed as additional pilot signals at the receiver for improved channel estimation. This innovative use of MGCC not only optimizes the identification and utilization of these carriers but also ensures that the selection of reliable data carriers remains unaffected. By transforming the high PAPR problem into a performance advantage, the scheme significantly reduces the computational complexity typically associated with separate PAPR reduction and channel estimation processes, making it particularly suitable for low-complexity wireless devices. The proposed approach determines peak-powered subcarriers entirely from the transmitted signal, eliminating the need for receiver-to-transmitter feedback and thereby simplifying system design without compromising performance. Furthermore, the study identifies optimal companding parameters to achieve an effective balance between error performance and PAPR reduction. Extensive simulations under Rayleigh and Rician fading channels with varying tap configurations demonstrate the robustness and versatility of the proposed scheme. Performance evaluations, including mean square error and bit-error-rate analyses, confirm the superiority of the proposed approach when paired with least square and linear minimum mean square error channel estimators. The impact of receive antenna correlation on error performance is also analyzed, revealing a nonlinear trend where low correlation levels have minimal effect, while variation in correlation influences system behavior. The results highlight consistent and reliable performance across diverse fading environments, underscoring the potential of the proposed scheme to enhance the efficiency and reliability of next-generation wireless communication systems.

## Introduction

Over the past decades, wireless communication systems have undergone significant advancements to meet the escalating demands for higher data rates, enhanced reliability, and increased network capacity^1,2^. Among the key enabling technologies, orthogonal frequency-division multiplexing (OFDM) has emerged as a fundamental multicarrier modulation (MCM) technique, widely adopted in 4G and 5G wireless networks due to its high spectral efficiency, robustness against multipath fading, and ability to support high data rates^3,4^. Complementing OFDM, multiple-input multiple-output (MIMO) technology has further revolutionized wireless systems by exploiting spatial diversity and multiplexing gains, leading to substantial improvements in spectral efficiency and system capacity^5,6^. However, despite these advantages, the practical deployment of MIMO-OFDM systems is constrained by the need for accurate channel estimation, which remains a fundamental challenge due to the dynamic nature of wireless fading environments and the complexity of multi-antenna processing^7,8^. Addressing this challenge is critical for ensuring reliable communication and maintaining optimal system performance in next-generation wireless networks.

Several channel estimation techniques have been extensively studied to enhance the performance of MIMO-OFDM systems, with least square (LS) and linear minimum mean square error (LMMSE) being the most widely used channel estimators^9,10^. The LS estimator does not require prior knowledge of channel statistics, making it computationally efficient; however, its accuracy is often suboptimal. In contrast, the LMMSE estimator leverages second-order channel statistics to minimize the mean square error (MSE), offering improved performance at the cost of increased computational complexity^11,12^. Irrespective of the channel estimation method, another challenge is to accurately estimate the wireless channel while minimizing the pilot overhead.

To address this, researchers have explored various data-aided channel estimation (DACE) schemes, categorized into iterative and non-iterative approaches. While non-iterative DACE schemes are susceptible to error propagation due to data detection inaccuracies, iterative DACE schemes often entail high computational complexity and increased communication latency^13^. To mitigate these issues, we first proposed a receiver-based DACE scheme for both single-input single-output (SISO) and MIMO-OFDM systems, which identifies the most reliable data carriers at the receiver^14^. Subsequently, we introduced an optimized pilot pattern for DACE in MIMO-OFDM systems, utilizing a single pilot subcarrier with maximum uniform pilot spacing to enhance spectral efficiency^15^. However, the receiver-based DACE scheme involves multiple distance calculations for each data carrier, imposing a computational burden on the system.

To overcome this limitation, we proposed a novel low-complexity peak power-assisted DACE scheme, which selects peak power carriers at the transmitter and employs them as additional pilot signals^16^. This approach eliminates the need for multiple distance calculations at the receiver, significantly reducing computational complexity. Furthermore, to mitigate the high peak-to-average power ratio (PAPR), we integrate a gamma correction companding (GCC) technique, ensuring seamless compatibility with the DACE framework. The proposed scheme effectively balances estimation accuracy, computational efficiency, and PAPR reduction, making it a promising solution for next-generation wireless systems.

This study introduces a novel transmitter-assisted scheme for DACE and PAPR reduction, designed to achieve accurate channel estimation and efficient PAPR mitigation with low computational complexity. The system’s performance is rigorously evaluated across diverse channel environments, specifically Rayleigh and Rician fading channels with varying numbers of channel taps. Rayleigh fading, characterized by the absence of a line-of-sight (LOS) path, results in significant signal fluctuations due to multiple reflections, diffractions, and scattering. Conversely, Rician fading, which includes a dominant LOS component alongside scattered paths, offers more stable signal propagation. To comprehensively assess the DACE scheme, we analyze three distinct channel conditions: favorable (Rician with strong LOS), moderate (Rician with moderate LOS), and challenging (Rayleigh fading). To address high PAPR, we employ the Modified Gamma Correction Companding (MGCC) technique, known for its robustness across various propagation conditions. By optimizing the companding parameters for each channel scenario, we achieve significant PAPR reduction, enhanced error performance, and improved channel estimation. The multi-parametric optimization of the companding scheme ensures adaptability to different channel conditions, providing greater flexibility. Our findings reveal that the proposed DACE scheme performs consistently well under both Rayleigh and Rician fading, provided an appropriate number of channel taps is selected for the given OFDM subcarriers. This demonstrates the adaptability and effectiveness of the DACE scheme in diverse channel conditions.

The main contributions of this study are as follows:

- A novel peak power-assisted DACE scheme is proposed to reduce the computational complexity of traditional receiver-based DACE schemes. Unlike traditional DACE schemes, the proposed scheme uses the peak power carriers as additional pilot signals. This innovative approach transforms the high PAPR challenge into a performance advantage by re-purposing high-peak power carriers for channel estimation, thereby reducing the computational burden on the receiver. Additionally, because the selection of peak-powered subcarriers relies solely on the transmitted signal’s characteristics, no receiver-to-transmitter feedback is needed, thereby simplifying system design.
- The MGCC technique is used to identify peak-powered carriers and mitigate high PAPR by compressing signal amplitudes at the transmitter and expanding them back at the receiver. This process lowers peak values, improves power amplifier efficiency, and minimizes distortion during transmission.
- Optimal companding parameters are determined for Rayleigh and Rician fading channels to reduce PAPR, improve error performance, and retain high peak-powered carriers for channel estimation. This achieves a balanced trade-off between PAPR reduction, channel estimation accuracy, and system flexibility.
- The proposed scheme is evaluated under various channel conditions using LS and LMMSE channel estimators. Simulation results are obtained for both SISO and MIMO-OFDM systems, considering different numbers of channel taps and Rician K-factor, with MSE and bit-error rate (BER) as the primary performance metrics. The results demonstrate the robustness and versatility of the proposed scheme across diverse fading environments.

Additionally, we investigate the effect of receive antenna correlation on error performance in MIMO systems. Our analysis demonstrates that while low correlation has a negligible impact, increasing correlation progressively degrades diversity gain, with fully correlated channels resembling a SISO system. The key novelty of this work lies in the integration of PAPR reduction and channel estimation into a single, computationally efficient framework. By leveraging high-peak power carriers as additional pilot signals through the MGCC technique, the proposed scheme not only mitigates the PAPR problem but also enhances channel estimation accuracy. This dual-functionality approach transforms a traditional challenge (high PAPR) into a performance advantage, significantly reducing the computational complexity typically associated with separate PAPR reduction and channel estimation processes. This innovation is particularly suited for modern wireless devices that demand low-complexity solutions, making it a promising advancement for next-generation communication systems.

The remainder of this paper is structured as follows: “Related works” provides a comprehensive overview of existing channel estimation schemes, along with studies that analyze system performance under various channel conditions. “System model” presents the system model and discusses the effect of multipath fading channels on the peak power-assisted DACE scheme. “Peak power-assisted DACE algorithm” delineates the proposed peak power-assisted DACE algorithm employing the MGCC technique. “Results and discussion” presents the simulation results, while “Conclusion” concludes the paper.

**Table 1:** Summary of related works on channel estimation methods and performance metrics for various wireless communication systems.

System configuration	Estimation methods/channel types	Performance metrics	Ref.
MIMO	LMMSE	MSE	^13^
SISO and MIMO-OFDM	LS and LMMSE	MSE, BER, and spectral efficiency (SE)	^14^
MIMO	LMMSE	MSE and BER	^17^
MIMO-OFDM	MMSE	MSE	^18^
SISO and MIMO-OTFS	LMMSE	MSE and symbol-error-rate (SER)	^19^
MIMO-OTFS	LMMSE	MSE, BER, and SE	^20^
MIMO-OFDM	MMSE	MSE and BER	^21^
Multicell large antenna systems	LMMSE	BER	^22^
OFDM	LS and MMSE	MSE and frame-error-rate (FER)	^23^
Massive MIMO	LS	FER	^24^
OFDM	LS	MSE and BER	^25^
MIMO	LMMSE	MSE and block-error-rate (BLER)	^26^
Multiuser systems	LMMSE	MSE	^27^
Deep-space communication system	CNN-LSTM, LS, and LMMSE	MSE	^28^
Deep-space communication system	ASG filtering, LS, and fixed-SG	MSE and BER	^29^
OFDM	Rayleigh and AWGN	BER	^30^
MIMO	Rayleigh, Rician, and Nakagami	MSE	^31^
MIMO-OFDM	Rayleigh, Rician, and AWGN	MSE, BER, and Throughput	^32^
Multiuser MC-CDMA	Rayleigh, Rician, and Nakagami	BER	^33^
M-ary QAM	AWGN, Rayleigh, Rician, and Nakagami	BER	^34^

## Related works

As mentioned above, researchers have proposed various DACE schemes to enhance channel estimation accuracy without compromising the spectral efficiency of wireless systems. In^13^, an LMMSE-based DACE scheme is presented for MIMO systems that selectively utilizes detected symbol vectors as additional pilot signals. Simulation results demonstrate that the proposed estimator enhances the detection performance compared to the traditional LMMSE channel estimator. In^14^, a novel DACE scheme is proposed for both SISO and MIMO-OFDM systems. The proposed scheme accurately detects the most reliable data carriers and significantly reduces the pilot overhead, thus enhancing the spectral efficiency of MIMO-OFDM systems. Similarly, an iterative DACE scheme using clustering and reinforcement learning (RL) techniques for MIMO systems is presented in^17^, which improves system performance especially at high signal-to-noise ratio (SNR).

In^18^, a non-iterative MMSE-based channel estimator is proposed for MIMO-OFDM systems, which significantly reduces system complexity by eliminating the need for iterative processing. However, due to data detection errors, non-iterative methods are susceptible to error propagation. To address this issue, iterative methods have been developed in^19–25^. In^19^, an iterative DACE scheme is introduced for both SISO and MIMO orthogonal time frequency space (OTFS) systems, utilizing affine-precoded superimposed pilots to improve channel estimation accuracy. Similarly^20^, presents an iterative channel estimation and data detection algorithm for MIMO-OTFS systems in high-mobility scenarios. Simulation results show that the proposed algorithm significantly improves system spectral efficiency and BER performance. In^21^, another iterative DACE scheme is introduced that uses soft-decision symbols as additional pilot signals. Similarly, in^22^, an iterative DACE scheme is proposed for multicell large antenna systems, utilizing partially decoded data for channel estimation. Furthermore^23^, presents an iterative turbo channel estimation technique for OFDM systems, where soft-decision symbols are used as pilot signals in each iteration.

In^24^, an iterative LS channel estimation algorithm is proposed for a massive MIMO turbo-receiver. This approach combines log-likelihood ratios (LLR) from a low-density parity-check (LDPC) decoder with an MMSE estimator to generate soft data symbols, which are then MMSE-weighted and combined with pilots for channel estimation. In^25^, an iterative DACE scheme for OFDM systems is introduced that involves controlled superposition of training sequences. This scheme provides a flexible trade-off between bandwidth efficiency and system performance. In^26^, a semi-data-aided LMMSE channel estimator for MIMO systems is proposed, which uses data symbols as pilot signals and effectively reduces the computational complexity of iterative methods. Furthermore^27^, presents two semi-blind channel estimation methods for multiuser systems that utilize Gaussian mixture models and provide computational advantages through parallel processing.

In addition to the aforementioned DACE schemes, several other DACE schemes have been discussed in the literature. However, to the best of our knowledge, none of them presents a joint PAPR reduction and peak power-assisted DACE scheme for MIMO-OFDM systems.

**Fig. 1:**
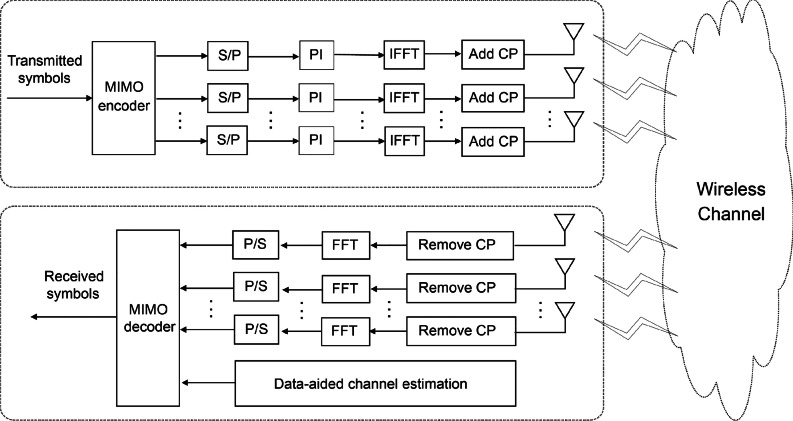
MIMO-OFDM system model.

Beyond conventional DACE schemes, several recent contributions focus on channel estimation for deep-space communications. For instance^28^, proposes a deep learning-based channel estimation framework for deep-space communications affected by solar scintillation and large Doppler shifts. By modeling the channel with a Gaussian Doppler spectrum and employing a hybrid convolutional neural network-long short-term memory (CNN-LSTM) architecture, the method effectively captures both spatial features and temporal variations in the channel. Simulation results show that the proposed method achieves improved MSE performance over the traditional LMMSE method and maintains strong robustness under low SNR and severe scintillation conditions. Similarly^29^, presents an adaptive Savitzky–Golay (ASG) filtering method for channel estimation in deep-space communication systems affected by strong Doppler shifts and solar scintillation. By adaptively adjusting filter parameters based on channel variation, the ASG method improves estimation accuracy compared to traditional LS and fixed-parameter SG filters. Simulation results demonstrate that the approach effectively tracks highly time-varying channels and enhances robustness under low SNR conditions.

Apart from above channel estimation methods, we have also reviewed works that analyze system performance under various channel conditions. In this respect^30^, evaluates the BER performance of an OFDM system under Rayleigh fading and additive white Gaussian noise (AWGN) channels. Simulation results show that the QPSK modulation scheme offers better performance than QAM modulation schemes. In^31^, two widely used channel estimation techniques, LS and MMSE, are analyzed for MIMO systems under Rayleigh, Rician, and Nakagami fading channels. Simulation results demonstrate that while both techniques improve system performance, MMSE outperforms LS, especially for Rician channels. Similarly^32^, evaluates the performance of the LS estimator in MIMO-OFDM systems under Rayleigh, Rician, and AWGN channels. In^33^, the BER performance of a multicarrier code division multiple access (MC-CDMA) system is analyzed under Rayleigh, Rician, and Nakagami fading channels. Moreover^34^, evaluates the BER performance of M-QAM modulation schemes under various channel conditions, including AWGN, Rayleigh, Rician, and Nakagami fading.

In addition to the above studies, numerous other studies have analyzed system performance under different channel conditions. However, to the best of our knowledge, none of them evaluates the performance of the DACE scheme under various channel conditions. The studies discussed above are comprehensively summarized in Table 1, which highlights their system configurations, channel estimation methods, performance metrics, and relevant references.

## System model

Figure 1 illustrates the transceiver architecture of a MIMO-OFDM system with $N_t$ and $N_r$ number of transmit and receive antennas. Using the DACE scheme, pilot insertion (PI) selects specific reference subcarriers within the transmitted data, enabling accurate channel estimation by mitigating distortions such as fading and noise. The inverse fast Fourier transform (IFFT) operations convert frequency-domain symbols into time-domain signals, while the fast Fourier transform (FFT) operations convert time-domain signals back into frequency-domain symbols. Furthermore, each IFFT block uses a cyclic prefix (CP) to provide protection against inter-symbol interference (ISI)^35^.

The received signal for a MIMO-OFDM system is given as:

1 $$\begin{aligned} \mathbf {y=Hx + z}, \end{aligned}$$

where the vector $\mathbf{x}$ of size $N_t\times 1$ represents the transmitted signal on the $\mathit{n}^{th}$ subcarrier, $\mathbf{z}$ is the complex AWGN vector with mean zero and covariance matrix $\mathbf{R}_{\mathbf{z}} = {\sigma _{\mathbf{z}}^2} \textbf{I}_{\mathit{N}}$, and matrix $\mathbf{H}$ of size $N_r\times N_t$ represents the channel frequency response (CFR) and is given as:

2 $$\begin{aligned} \mathbf{H} = \begin{bmatrix} H_{1 1} & \cdots & H_{1 N_t} \\ \vdots & \ddots & \vdots \\ H_{N_r 1} & \cdots & H_{N_r N_t} \end{bmatrix}. \end{aligned}$$

In matrix-vector form, the received signal is also given as:

3 $$\begin{aligned} \mathbf {y=Ch+z}, \end{aligned}$$

where matrix $\mathbf{C} = \sqrt{N} \mathbf {diag(x)F}$, $\mathbf {diag(x)}$ is an $N \times N$ diagonal matrix hosting the transmitted symbols, $\mathbf{F}$ is the partial FFT matrix consisting of the first *L* columns of the full FFT matrix, and $\mathbf{h}$ represents the *L*-tap channel impulse response (CIR) with tap coefficients $[\mathit{h_0, h_1, \ldots , h_{L-1}}]^\top$.

### Effect of channels on peak power-assisted DACE

In this work, we considered multipath fading channels to evaluate the proposed algorithm. A multipath Rayleigh fading channel is time-varying with multiple paths caused by scattering, diffraction, or reflection, making accurate channel estimation essential for reliable communication. Additionally, we examined Rician fading, which models environments with a strong LOS path alongside scattered components, such as rural wireless communication and indoor scenarios with clear LOS. Rayleigh fading is a special case of Rician fading where the K-factor $K = 0$, indicating the absence of an LOS component, leaving only scattered signals. In this case, the Rician distribution simplifies to the Rayleigh distribution.

As the Rician factor $K$ increases, the power of the LOS component increases, reducing the effect of multipath fading. This leads to fewer deep fades and improved signal quality, thereby lowering the BER. In the limit, as $K \rightarrow \infty$, the channel approximates an AWGN channel where the BER approaches its minimum. Conversely, in Rayleigh fading ($K = 0$) there is no dominant LOS component, resulting in severe multipath fading, characterized by deeper fades and higher BER.

Channel estimation is more challenging in Rayleigh fading due to rapid and random channel fluctuations. The channel response, modeled as a complex Gaussian random variable, increases the MSE in estimation. In Rician fading, the presence of a stable LOS component, represented by $h_{\text {ric}} = h_{\text {LOS}} + h_{\text {NLOS}}$, enables more accurate tracking and estimation, reducing the MSE.

The Rician $K$-factor, which is the ratio of the LOS power to the scattered power $\left( K = \frac{A^2}{2\sigma ^2} \right)$, simplifies peak power-based channel estimation. Higher values of $K$ result in more higher power peaks, making the estimation process easier. In contrast, in Rayleigh fading, the absence of an LOS component leads to fluctuating power levels, which complicates peak power-based methods. Thus, in Rician fading, the stable LOS power improves BER performance and reduces MSE, while the scattered nature of Rayleigh fading results in higher BER and more difficult estimation.

In both fading scenarios, optimization of the companding parameters is critical for signal integrity. In Rician fading, with a strong LOS component, companding can be adjusted to maximize PAPR gain, thereby improving signal robustness. In Rayleigh fading, due to the rapid channel variations, adjustments may focus more on maintaining consistent error performance rather than PAPR gain. Therefore, effective companding strategies depend on balancing PAPR and error performance based on the specific fading environment.

**Fig. 2:**
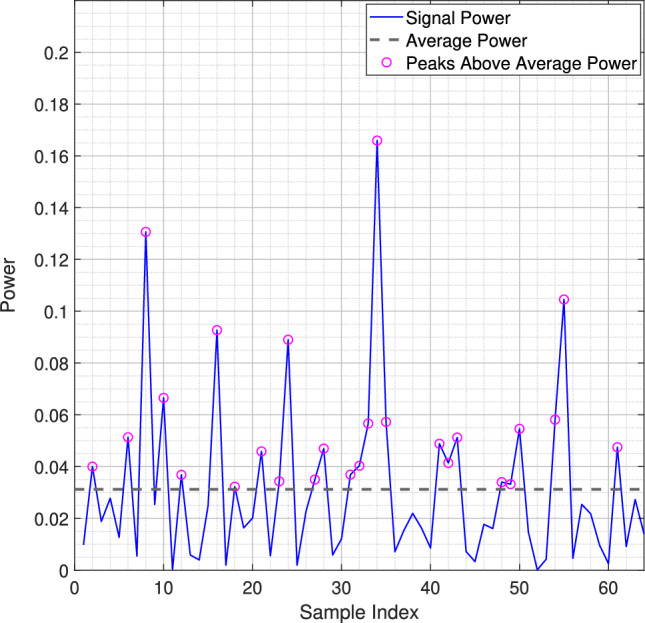
A random OFDM symbol with *N* = 64 shows that some subcarriers can exhibit much higher power peaks than the signal’s average power.

## Peak power-assisted DACE algorithm

In our previous work, we proposed a novel peak power-assisted DACE algorithm using the GCC technique to mitigate high PAPR^16^. We now employ the MGCC technique, which is robust to diverse channel conditions. The peak power-assisted DACE algorithm using the MGCC technique is discussed as follows:

In OFDM, data is transmitted on a number of subcarriers spaced at regular intervals. A large number of independently modulated subcarriers are added together to form an OFDM symbol. The orthogonality of these subcarriers is analyzed using the autocorrelation function^36^. If the orthogonality of these subcarriers is not maintained and they are in phase, they can constructively interfere with each other, resulting in high peak power levels.

Mathematically, the OFDM transmitted signal is given as^37^:

4 $$\begin{aligned} s(t) = \sum _{n=0}^{N-1} x_n(t) = \frac{1}{\sqrt{N}} \sum _{n=0}^{N-1} X_n e^{j 2 \pi f_n t}, \end{aligned}$$

where *N* is the total number of OFDM subcarriers, *x*$_n$(*t*) represents the waveform of the $\textit{n}$th subcarrier, *X*$_n$ represents the data on the $\textit{n}$th subcarrier, and $f_n$ is the frequency of the $\textit{n}$th subcarrier. The instantaneous power of this signal is: $|s(t)|^2$ = *s*(*t*)*s*$^*$(*t*), where *s*$^*$(*t*) is the complex conjugate of *s*(*t*).

Now, the PAPR is defined as: max($|s(t)|^2$) / E[$|s(t)|^2$], where max($|s(t)|^2$) represents the peak power (maximum instantaneous power), and E[$|s(t)|^2$] represents the average power (expected value of the instantaneous power). Fig. 2 illustrates the high PAPR phenomenon, showing that while the average power remains stable, the instantaneous power can have significantly high peaks. Although high PAPR is typically a drawback in OFDM systems, increasing the complexity of analog-to-digital and digital-to-analog conversions and reducing power amplifier efficiency, we exploit this effect to identify the most reliable data carriers for the DACE scheme. To achieve this, we compute the peak power of each subcarrier, sort them in descending order, and then select the top $N_{rsc}$ subcarriers from the sorted list to serve as additional pilot signals for the DACE scheme. The occurrence of high-powered subcarriers in the OFDM signal results from constructive interference, regardless of whether they carry data or pilot symbols. The selection of peak-powered subcarriers is based on the transmitted signal’s characteristics. Therefore, no additional feedback from the receiver to the transmitter is required, simplifying the system design.

### Modified gamma correction companding technique

Researchers have proposed various methods to mitigate high PAPR, including clipping, filtering, linear precoding, coding schemes, phase optimization, nonlinear companding, tone reservation, tone injection, constellation shaping, selective mapping, and partial transmit sequences. However, in this work, the goal is to achieve a high PAPR reduction while simultaneously improving system performance and enabling the detection of high-peak carriers for reliable channel estimation. To achieve this, we employ MGCC, a nonlinear companding technique with multiple optimized parameters, providing flexibility under varying channel conditions.

Traditional companding schemes, such as A-law and $\mu$-law, offer limited flexibility because they rely on only a few tuning parameters. In contrast, the MGCC technique introduces an additional amplitude-control parameter together with the companding factor $\gamma$. This added degree of freedom allows the companding profile to be more precisely shaped under different wireless fading conditions, enabling a better balance between PAPR reduction, BER performance, and channel estimation. Since Rayleigh and Rician channels introduce different distortion characteristics, this flexibility is essential for maintaining consistent behavior across channel types. Moreover, MGCC can compress large peaks and expand lower-amplitude samples more effectively than conventional GCC, which improves both PAPR reduction and the reliability of high-peak carrier selection for channel estimation. After the MGCC expansion at the receiver, these peak carriers play a crucial role as reliable data points for channel estimation. To ensure this functionality, the companding parameters, particularly $\gamma$, must be carefully optimized so that peak values are not excessively compressed. Over-compression would yield significant PAPR reduction but eliminate the peaks needed for accurate channel estimation. Therefore, selecting these parameters involves achieving a trade-off between error performance, channel estimation accuracy, and PAPR gain.

**Fig. 3:**
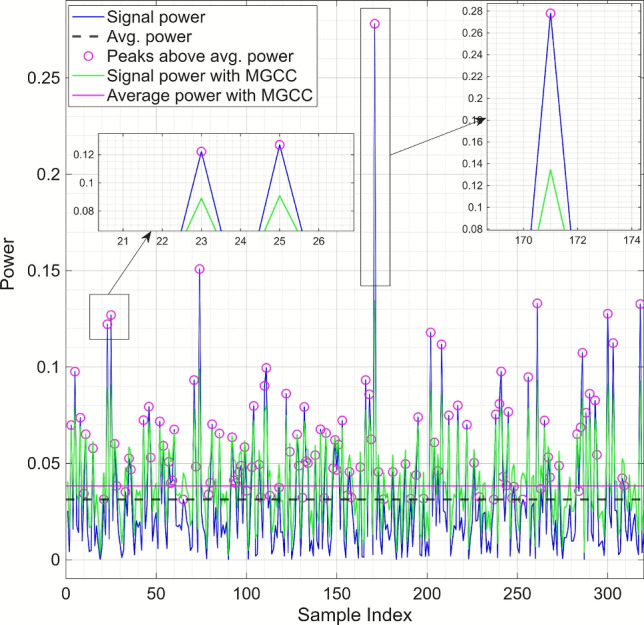
Effect of MGCC (A = 3, $\gamma$ = 2.5) on a regular OFDM signal: Larger peaks are compressed while smaller peaks are expanded, lowering dynamic range and average peak power.

Figure 3 illustrates the effect of MGCC on a regular OFDM signal, where larger peaks are compressed and smaller peaks are expanded. This reduces the signal’s dynamic range and lowers the average peak power, resulting in improved efficiency and reduced distortion.

Using a companding scheme with more tuning parameters enhances channel estimation accuracy by providing flexibility in signal compression, reducing distortion, and optimizing PAPR and BER trade-offs. This makes MGCC a robust choice for peak power-assisted estimation in both Rician and Rayleigh fading environments. By allowing the companding profile to be fine-tuned to specific channel conditions, MGCC effectively handles both high and low signal amplitudes. In Rician fading, it optimizes signal integrity and improves SNR, while in Rayleigh fading it improves noise immunity for weaker signals, facilitating accurate channel estimation.

It’s worth noting that MGCC maintains a similar flat profile at lower $A$ (around 1.0), but the profile can be reshaped to avoid flat regions and improve error performance. By choosing an appropriate value of $A$, a significant reduction in PAPR can be achieved. The degree of companding can then be controlled using $\gamma$ to achieve the desired PAPR reduction. In addition, the overall profile can be further adjusted to improve error performance. The MGCC function together with its inverse (expanding), can be expressed as^38^:

5 $$\begin{aligned} H_\gamma (x) = \text {sgn}(x) \frac{[A |x|] ^ \gamma }{1 + |x| ^ \gamma }, \end{aligned}$$

and

6 $$\begin{aligned} H^{-1}_\gamma (y) = \text {sgn}(y) \left[ \frac{A}{|y|} - 1\right] ^{-\frac{1}{\gamma }}. \end{aligned}$$

**Table 2:** Computational complexity and error performance of various state-of-the-art channel estimation techniques.

Channel estimation method	Error performance	Complexity	Ref.
Receiver-based DACE scheme	Superior than traditional DACE schemes	${\mathcal {O}(N \cdot M + N \log N)}$	^14^
Clustering and RL-based DACE scheme	Approaches to perfect CSI at high SNR	$\mathcal {O}(I_{\textit{EM}} \cdot G \cdot N + I \cdot N_t^3)$	^17^
ML-based adaptive CE algorithm	Better than conventional CE methods	$\mathcal {O}(I\cdot N^3)$	^39^
Variable pilot-assisted CE method	Improved for AWGN channel	$\mathcal {O}(N \cdot P+N\log N)$	^40^
EEVD-based CE method	Significantly improved at high SNR	$\mathcal {O}(N^3+N\log N)$	^41^
Semi-blind CE and pilot allocation method	Superior than classical CE methods	$\mathcal {O}(N^3)$	^42^
DL-based CE scheme	Outperforms CNN-based estimators	${\it{K}}_{\it{on}}^2 + 39946 {\it{K}}_{\it{on}} + 6336$	^43^
Transmitter-assisted DACE scheme	Equivalent to Receiver-based DACE	$\approx \mathcal {O}(N \log N)$	This work

*CE* channel estimation, *I*$_{{EM}}$ number of iterations for expectation-maximization (EM) algorithm, *I* number of iterations for CE, *G* number of clusters, *CSI* channel state information, *ML* maximum likelihood, *EEVD* enhanced eigenvalue decomposition, *DL* deep learning, *K*$_{on}$ number of active subcarriers, *CNN* convolutional neural network.

The companding parameter $\gamma$ determines the extent of companding, while *A* is optimally chosen to maximize SNR. It’s important to note that $H_\gamma (x)$ exhibits asymptotic behavior in terms of *x*, i.e., $\lim _{|x| \rightarrow \infty } H_\gamma (x) = A^\gamma$. Thus, as *x* approaches infinity, $H_\gamma (x)$ approaches $A^\gamma$ without actually reaching it. The maximum amplitude of the companded signal is controlled by adjusting *A* accordingly. As *x* approaches infinity, Eq. (5) becomes:

7 $$\begin{aligned} H_\gamma (x) = \frac{[A |x|]^\gamma }{1 + |x|^\gamma } = \frac{1}{[A |x|]^{-\gamma } + A^{-\gamma }} \approx A^\gamma |_{x \rightarrow \infty }. \end{aligned}$$

Since $H_\gamma (x)$ approaches a horizontal asymptote at $A^\gamma$, any discontinuity in the signal will not affect its distribution. In conclusion, $\gamma$ determines the degree of companding.

Using Rapp’s model of the solid-state power amplifier (SSPA), which provides an effective approximation, the amplitude/amplitude (AM/AM) and amplitude/phase (AM/PM) characteristics of an SSPA can be expressed as^38^:

8 $$\begin{aligned} T(|x(t)|) = \frac{g |x(t)|}{\left[ 1 + \left( \frac{g|x(t)|}{A_{\text {sat}}}\right) ^{2k}\right] ^{\frac{1}{2k}}}, \quad \Phi (|x(t)|) = 0 \, \text {(rad)}, \end{aligned}$$

where $T(\cdot )$ and $\Phi (\cdot )$ denote the AM/AM and AM/PM transformations, respectively. It should be noted that the SSPA only performs the AM/AM transformation with a gain $g$. The parameter $A_{\text {sat}} = \sqrt{P_{\text {sat}}}$ represents the output saturation amplitude, where $P_{\text {sat}}$ is the saturated output power. To avoid nonlinear distortion, the SSPA must operate in the linear region, where the knee factor $k$ controls the smoothness of the transition from the linear to the saturation region. The output of the SSPA is given as:

9 $$\begin{aligned} y(t) = T(|x(t)|) e^{j\{\theta (x(t)) + \Phi (x(t))\}}. \end{aligned}$$

Finally, for both LS and LMMSE channel estimation methods, the peak power carriers are incorporated with known pilot symbols to refine the initial channel estimates. For *p* as pilot and *r* as reliable data indices, Eq. (3) is updated as:

10 $$\begin{aligned} \mathbf{y}_{rp}=\mathbf{C}_\mathit{rp}\mathbf{h}+\mathbf{z}_\mathit{rp}, \end{aligned}$$

where

11 $$\begin{aligned} \mathbf{C}_\mathit{rp} = \sqrt{N} \mathbf{diag}(\mathbf{x}_{\mathit{rp}})\mathbf{F}_\mathit{rp}. \end{aligned}$$

Now, the transmission vector, partial FFT matrix, and observation vector are respectively given as:

12 $$\begin{aligned} \mathbf{x}_{\mathit{rp}}= & \begin{bmatrix} \mathbf{x}(p) \\ \mathbf{x}(r) \end{bmatrix}, \end{aligned}$$

13 $$\begin{aligned} \mathbf{F}_\mathit{rp}= & \begin{bmatrix} \mathbf{F}(p) \\ \mathbf{F}(r) \end{bmatrix}, \end{aligned}$$

14 $$\begin{aligned} \mathbf{y}_{\mathit{rp}}= & \begin{bmatrix} \mathbf{y}(p) \\ \mathbf{y}(r) \end{bmatrix}. \end{aligned}$$

Consequently, the LS and LMMSE channel estimates, using both pilots and peak power carriers to re-estimate the wireless channel, are given by the following expressions:

15 $$\begin{aligned} \hat{\mathbf{h}}_{rp}^{(\text {LS})}= & \left( \mathbf{C}_\mathit{rp}^{ H } \mathbf{C}_\mathit{rp}\right) ^{-1}\mathbf{C}_\mathit{rp}^{ H }\mathbf{y}_\mathit{rp}, \end{aligned}$$

16 $$\begin{aligned} \hat{\mathbf{h}}_\mathit{rp}^{(\text {L M M S E})}= & \left( \mathbf{R}_{\mathbf{h}}^{-1}+\mathbf{C}_\mathit{rp}^{ H }\mathbf{R}_{\mathbf{z}}^{-1}\mathbf{C}_{\mathit{rp}}\right) ^{-1}\mathbf{C}_{\mathit{rp}}^{ H }\mathbf{R}_{\mathbf{z}}^{-1} \mathbf{y}_{\mathit{rp}}. \end{aligned}$$

By leveraging peak power carriers as additional pilot signals, the proposed transmitter-assisted DACE scheme improves system MSE and BER performance. This preserves channel estimation accuracy while significantly reducing the computational complexity of traditional receiver-based DACE schemes by a factor of *M*, where *M* represents the size of the constellation set^16^. Table 2 provides a comprehensive comparison of computational complexity and error performance for various state-of-the-art channel estimation techniques. The table highlights the trade-offs between accuracy and complexity across different methods, demonstrating the competitive performance and computational efficiency of the proposed transmitter-assisted DACE scheme. Given the low computational complexity and high accuracy, the proposed channel estimation scheme is well-suited for various real-world applications, including intelligent transportation systems (ITS) and vehicle-to-everything (V2X) communication, where efficient and reliable communication is essential for real-time data exchange, safety, and seamless connectivity in dynamic environments. Algorithm 1 represents the pseudocode for the proposed transmitter-assisted DACE scheme with MGCC.

**Fig. 4:**
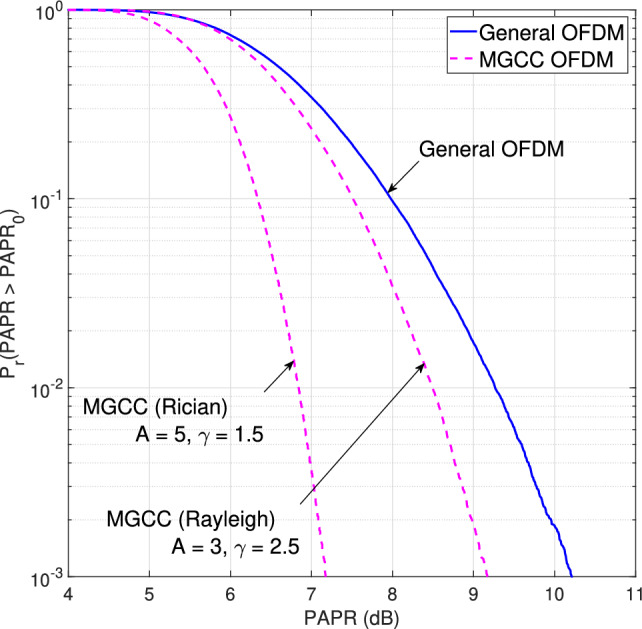
CCDF curves for general OFDM and MGCC-OFDM.

**Algorithm 1:**
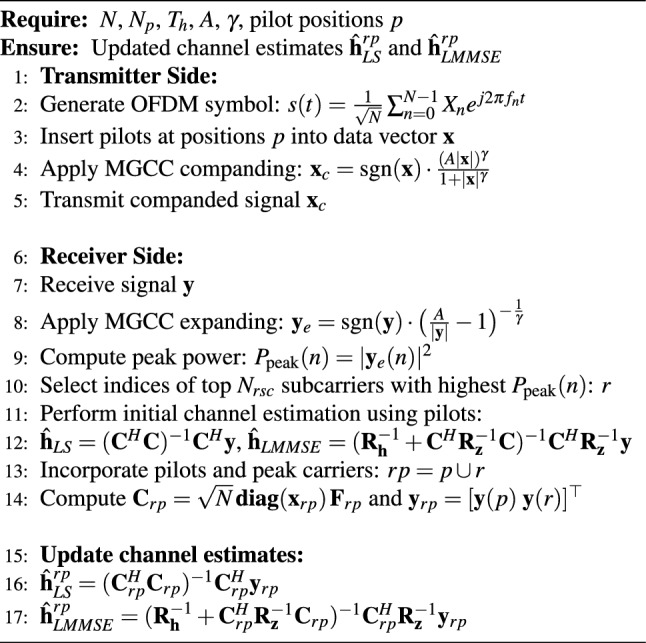
Transmitter-assisted DACE with MGCC.

## Results and discussion

This section presents the simulated results obtained using the parameters listed in Table 3. Using different numbers of channel taps for both Rayleigh and Rician fading channels, the MSE performance of the proposed transmitter-assisted DACE scheme is evaluated for both LS and LMMSE channel estimators in a SISO system. As mentioned in “Modified gamma correction companding technique”, MGCC handles both high and low signal amplitudes by optimizing the companding parameters for specific channel conditions. Fig. 4 illustrates the complementary cumulative distribution function (CCDF) curves for ‘General OFDM’ and ‘MGCC OFDM’. The CCDF represents the probability $P(PAPR>PAPR_0)$, where the PAPR exceeds a given threshold $PAPR_0$. The CCDF curves confirmed significantly lower PAPR probabilities compared to General OFDM. We determined the optimal parameters for Rayleigh (A = 3, $\gamma$ = 2.5) and Rician (A = 5, $\gamma$ = 1.5) channels, ensuring comparable MSE and BER in both fading environments. These parameters are used for all subsequent simulated graphs.

**Table 3:** Simulation parameters.

Parameter	Specification
Number of OFDM subcarriers	$N = 256$
Number of pilot subcarriers	$N_p = 16$
Number of data subcarriers	$N_d = 240$
Channel model	Rayleigh and Rician fading
Number of channel taps	$L = 16, 8,$ and 4
Pilot pattern	Comb-type
Channel estimation	DACE scheme
Channel estimation methods	LS and LMMSE
Modulation schemes	BPSK, 4QAM, and 8PSK
Knee factor (*k*) in (8)	0.4
MIMO configurations	$1\times 2$, $2\times 4$, and $2\times 8$

**Fig. 5:**
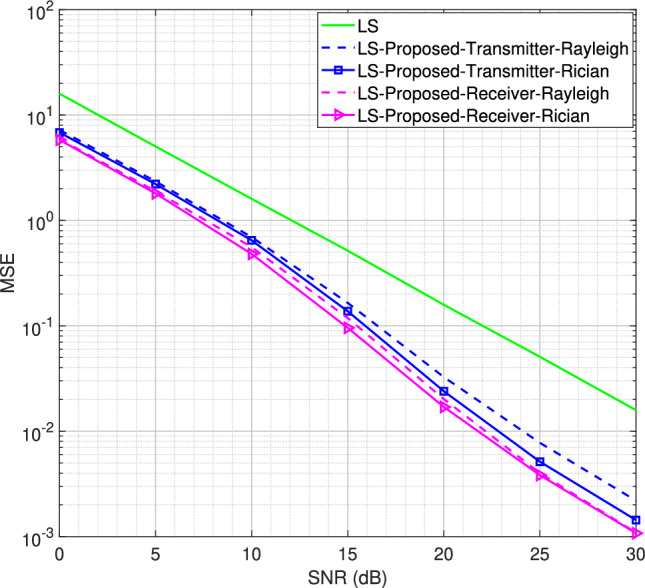
MSE performance for the SISO system using LS channel estimator for BPSK modulation scheme, $L = 16$.

**Fig. 6:**
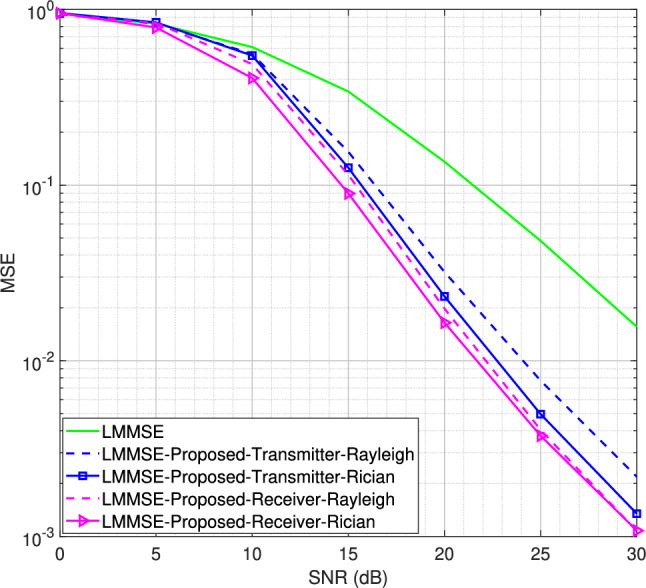
MSE performance for the SISO system using LMMSE channel estimator for BPSK modulation scheme, $L = 16$.

**Fig. 7:**
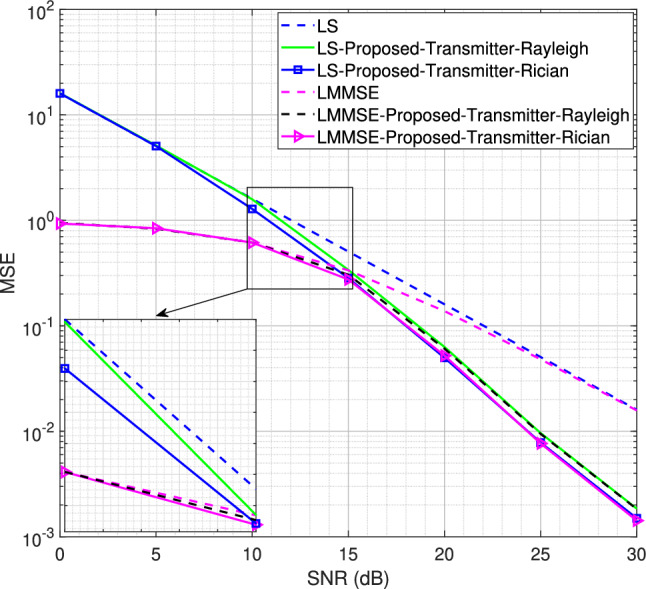
MSE performance for the SISO system using transmitter-assisted DACE scheme for 4QAM modulation scheme, $L = 8$.

Starting with *L* = 16 number of channel taps and a Rician factor *K* = 10, the MSE performance for LS and LMMSE estimators using the BPSK modulation scheme is shown in Figs. 5 and 6, respectively. It is observed that both the transmitter-assisted and receiver-based DACE schemes outperform the traditional LS and LMMSE channel estimators. However, the receiver-based DACE scheme (proposed in^14^) achieves slightly better performance than the transmitter-assisted DACE scheme under both Rayleigh and Rician fading channel conditions. Furthermore, the MSE performance of both DACE schemes for the Rayleigh fading channel is approximately equivalent to their performance for the Rician fading channel, demonstrating that the DACE scheme performs equally well under both fading conditions.

**Fig. 8:**
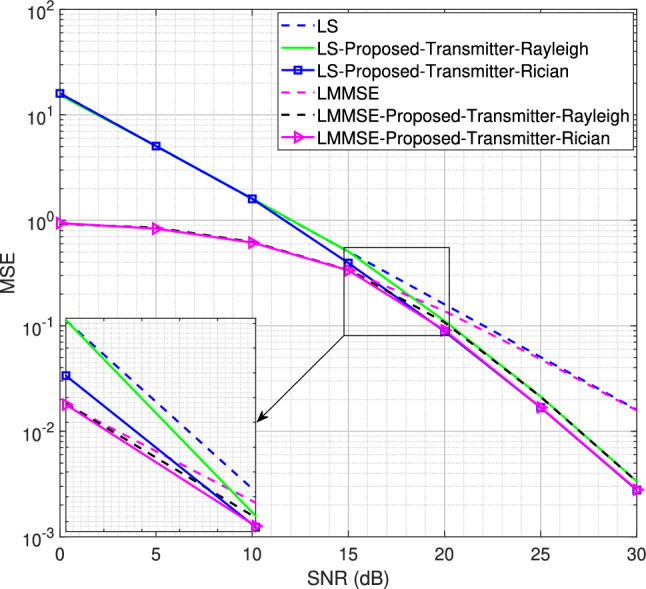
MSE performance for the SISO system using transmitter-assisted DACE scheme for 8PSK modulation scheme, $L = 8$.

**Fig. 9:**
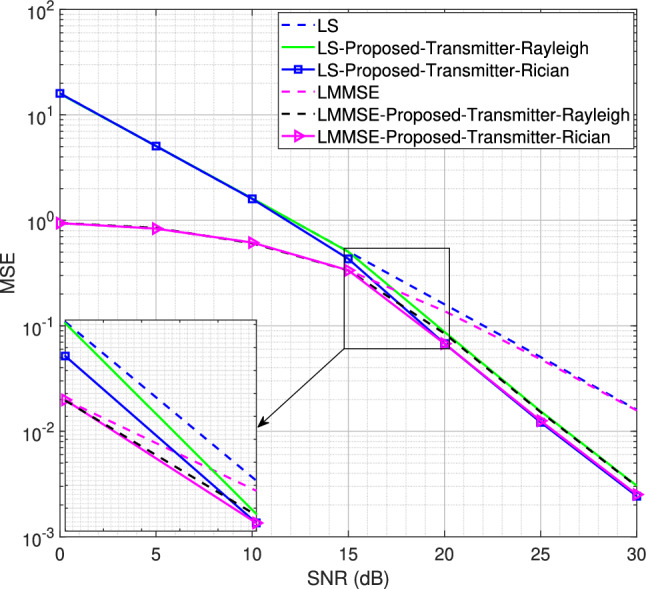
MSE performance for the SISO system using transmitter-assisted DACE scheme for 4QAM modulation scheme, $L = 4$.

We now reduce the number of channel taps from 16 to 8 to analyze the system performance for the proposed transmitter-assisted DACE scheme under both Rayleigh and Rician fading channel conditions. Using a strong LOS component with Rician factor *K* = 10, the MSE performance for the 4QAM and 8PSK modulation schemes is shown in Figs. 7 and 8, respectively. It is observed that the system MSE performance deteriorates drastically at low SNR for both LS and LMMSE channel estimators. Although the performance degradation occurs for both Rayleigh and Rician fading channels, the system MSE performance for the Rician fading channel is slightly better than that of the Rayleigh fading channel at low SNR. For 4QAM, the MSE performance improves from 5 dB to 30 dB for the Rician fading channel and from 10 dB to 30 dB for the Rayleigh fading channel. Similarly, in Fig. 8, the MSE performance for 8PSK with the Rician fading channel improves from 10 dB to 30 dB, while for the Rayleigh fading channel it improves from 15 dB to 30 dB. This indicates that the DACE scheme attains slightly better performance at low SNR for the Rician fading channel, while at high SNR its performance is almost the same for both Rayleigh and Rician fading channels. Furthermore, the 4QAM modulation scheme achieves better performance than 8PSK modulation scheme because it is easier to detect reliable data carriers with lower-order modulation schemes.

**Fig. 10:**
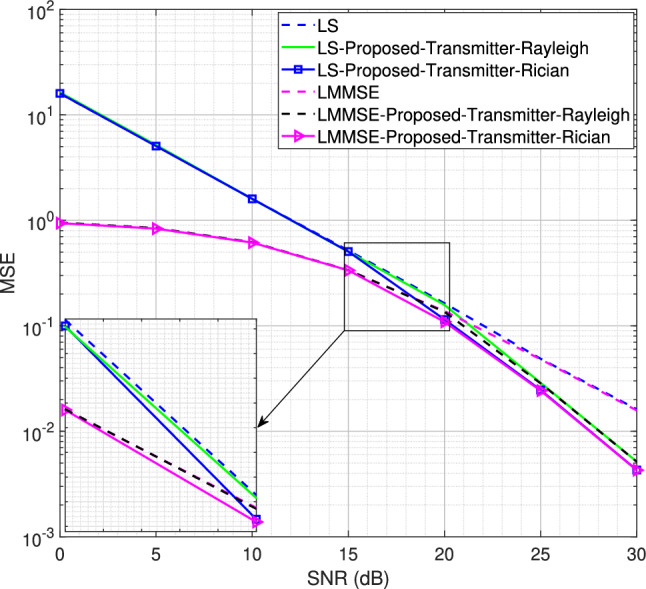
MSE performance for the SISO system using transmitter-assisted DACE scheme for 8PSK modulation scheme, $L = 4$.

Next, we reduce the number of channel taps from 8 to 4 while using the same Rician factor *K* = 10 for the proposed transmitter-assisted DACE scheme. The MSE performance for the 4QAM and 8PSK modulation schemes are shown in Figs. 9 and 10, respectively. It is worth noting that as the number of channel taps decreases, the MSE performance further deteriorates at low SNR for both Rayleigh and Rician fading channels. However, for 4QAM, the MSE performance improves from 10 dB to 30 dB for the Rician fading channel and from 15 dB to 30 dB for the Rayleigh fading channel. Similarly, for 8PSK, the performance improvement occurs from 15 dB to 30 dB for the Rician fading channel, while it occurs from 20 dB to 30 dB for the Rayleigh fading channel. This shows that regardless of the channel conditions, when the number of channel taps is reduced, the system experiences significant performance degradation at low SNR. The number of channel taps, i.e., the number of multipath components, is therefore of paramount importance in achieving the desired performance with the DACE scheme.

**Fig. 11:**
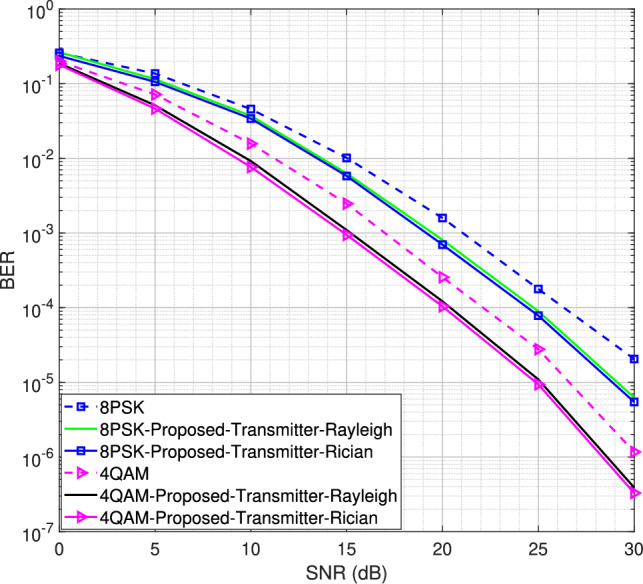
BER performance for the SIMO system using LS/LMMSE channel estimator for 4QAM and 8PSK modulation schemes, $L = 16$.

**Fig. 12:**
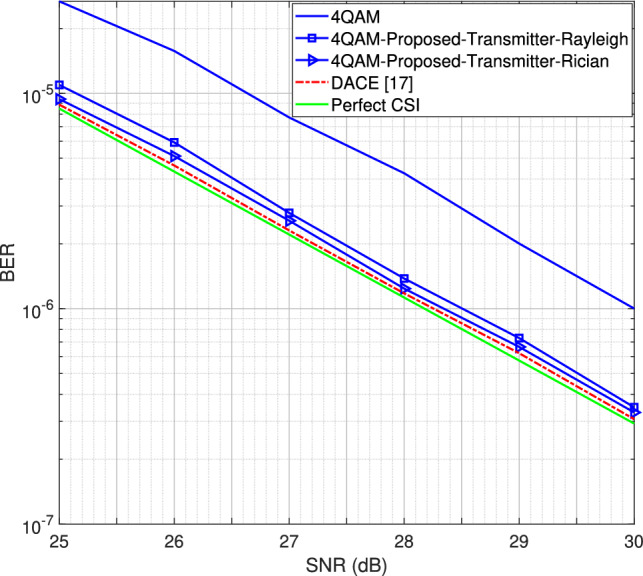
Performance analysis of the proposed transmitter-assisted DACE scheme using the 4QAM modulation scheme.

**Fig. 13:**
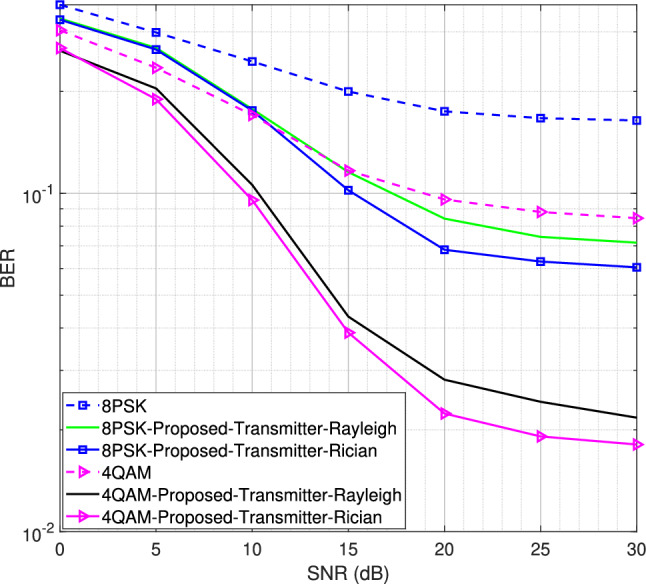
BER performance for $2\times 4$ MIMO configuration using LS/LMMSE channel estimator for 4QAM and 8PSK modulation schemes, $L = 16$.

We now evaluate the BER performance under Rician fading channel conditions for different MIMO configurations, including the $1\times 2$ single-input multiple-output (SIMO) system, as well as the $2\times 4$ and $2\times 8$ MIMO systems. The BER is defined as the number of error bits at the receiver divided by the total number of transmitted bits. The results, obtained for both 4QAM and 8PSK modulation schemes, are compared with the BER performance under Rayleigh fading channel conditions for the DACE scheme, as presented in^16^. Using *L* = 16 number of channel taps and a moderate LOS component with a Rician factor *K* = 5, the BER performance for the SIMO system is shown in Fig. 11. It is observed that the proposed transmitter-assisted DACE scheme outperforms traditional channel estimators under both Rayleigh and Rician fading channel conditions. However, it achieves comparable BER performance under both fading conditions.

Next, we compare the BER performance of the proposed transmitter-assisted DACE scheme with the DACE scheme developed in^17^. As mentioned earlier^17^, introduces an iterative, receiver-based DACE scheme that enhances the performance of the LMMSE channel estimator by using clustering and RL techniques. It employs the EM algorithm to compute the a-posteriori probabilities (APPs) of the received signals. By setting a threshold for the APPs, the proposed algorithm accurately detects symbols that are less distorted by noise and utilizes them as additional pilot signals to improve channel estimation accuracy. In this regard, the authors demonstrated that the performance of the proposed estimator approaches perfect CSI at high SNR, specifically in the range of 24 dB to 30 dB.

For comparison, we plotted the perfect CSI curve for the BER performance shown in Fig. 11 for the 4QAM modulation scheme. Fig. 12 presents the high SNR region (25 dB to 30 dB) of Fig. 11. The results demonstrate that while the BER performance of our proposed scheme also approaches perfect CSI at high SNR, the DACE scheme in^17^ achieves better performance than our proposed scheme but at the cost of significantly higher computational complexity, as shown in Table 2. Our proposed scheme avoids multiple distance calculations at the receiver to identify the most reliable data carriers for the DACE scheme. It efficiently identifies peak the power carriers and utilizes them as additional pilot signals, thereby significantly reducing system complexity.

**Fig. 14:**
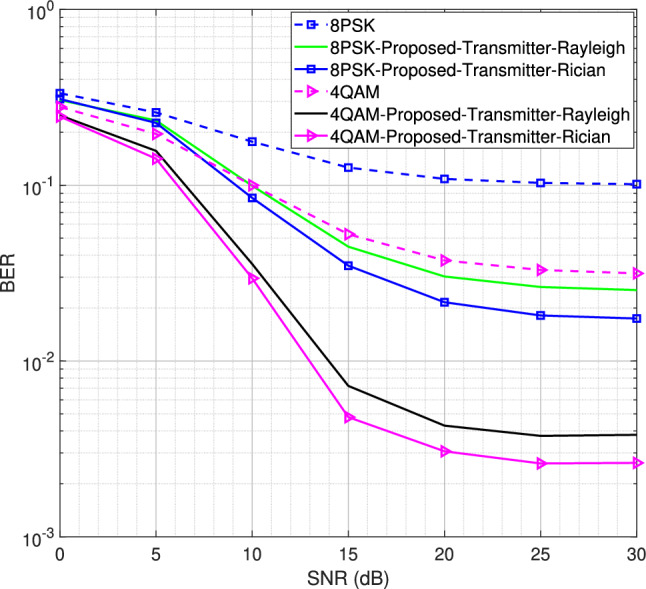
BER performance for $2\times 8$ MIMO configuration using LS/LMMSE channel estimator for 4QAM and 8PSK modulation schemes, $L = 16$.

Next, using *L* = 16 number of channel taps, we increase the Rician factor from 5 to 10 for the $2\times 4$ and $2\times 8$ MIMO configurations, as shown in Figs. 13 and 14, respectively. Similar behavior is observed for both configurations. However, the $2\times 8$ MIMO configuration demonstrates better performance than the $2\times 4$ MIMO configuration due to the diversity gain. The MSE and BER curves show that both the transmitter-assisted and receiver-based DACE schemes can enhance system performance over the entire range of SNR values, provided that a sufficient number of channel taps is utilized for the given OFDM subcarriers. Additionally, the proposed transmitter-assisted DACE scheme achieves equivalent MSE and BER performance compared to the receiver-based DACE schemes while significantly reducing computational complexity. Furthermore, better MSE and BER performance is observed at high SNR, as data equalization/channel estimation experiences less distortion at high SNR and vice versa.

We have also analyzed system performance using different pilot patterns employed in^15^. However, the proposed transmitter-assisted DACE scheme demonstrates behavior similar to that of the receiver-based DACE scheme and achieves maximum performance improvement with only an optimal comb-type pilot pattern designed in^15^. The proposed pilot pattern utilizes a single pilot subcarrier with maximum uniform pilot spacing, which is maintained with the help of reliable data carriers/peak power carriers identified between the pilot subcarriers. Knowing that only an optimal comb-type pilot pattern enables consistent performance improvement for both the transmitter-assisted and receiver-based DACE schemes, the simulated results presented above are obtained using this pilot pattern.

**Fig. 15:**
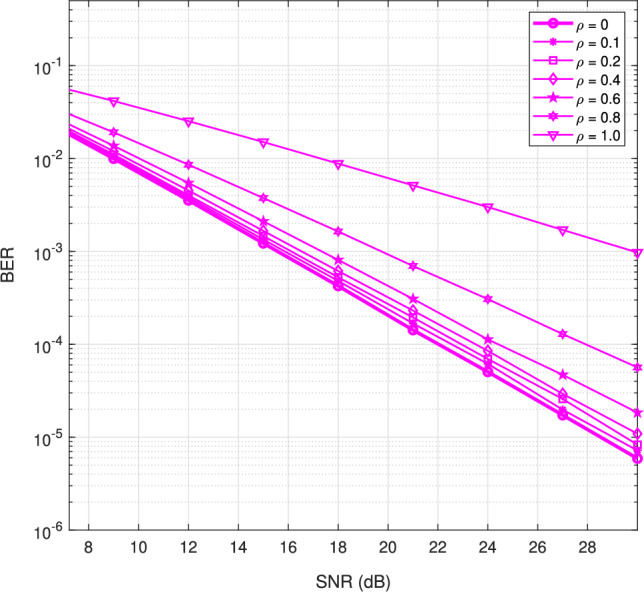
BER performance of the proposed transmitter-assisted DACE scheme under varying channel correlation conditions.

In a multipath wireless environment, studying the impact of channel correlation on error performance is crucial. When the two receive antennas experience uncorrelated fading, diversity techniques such as maximal ratio combining or selection combining significantly enhance error performance. However, in practical scenarios, receive antennas are often correlated due to physical constraints, such as limited spacing, or environmental factors, which ultimately degrade the diversity gain.

This correlation effect is particularly relevant in modern wireless systems, including Wi-Fi, LTE, and 5G, where device size limitations often lead to correlated antenna elements. As correlation increases, the advantage of independent fading diminishes, resulting in degraded BER performance. In extreme cases, where the channels become fully correlated, the system effectively loses its diversity gain and behaves similarly to a 1$\times$1 SISO system.

Figure 15 illustrates the impact of fading correlation on error performance across several correlation levels. The results indicate that system performance deteriorates in a nonlinear manner as correlation increases. For low correlation coefficients (ρ), such as 0.1 to 0.3, the degradation is minimal and can be largely ignored. However, as the correlation index increases, the deterioration in performance becomes more pronounced, following a monotonic trend.

Similarly, other channel estimation techniques, such as the ML-based adaptive algorithm proposed in^39^, also employ a recursive approach to achieve BER performance comparable to perfect CSI, which significantly increases system complexity. Therefore, most existing channel estimation techniques improve error performance at the cost of higher computational complexity. In contrast, our proposed scheme offers a novel low-complexity alternative to conventional receiver-based DACE schemes. Its ability to select peak power carriers and exploit them as additional pilot signals, combined with the MGCC technique for PAPR reduction, ensures robust and accurate channel estimation in diverse and challenging environments.

## Conclusion

In this paper, we presented a novel transmitter-assisted DACE scheme for both SISO and MIMO-OFDM wireless systems, demonstrating its effectiveness across various channel conditions through comprehensive performance analysis. The proposed DACE scheme achieves high-quality channel estimation with significantly reduced computational complexity, effectively addressing two critical challenges in modern wireless systems, PAPR reduction and accurate channel estimation, within a unified framework. By accurately selecting peak power carriers and repurposing them as additional pilot signals, the scheme transforms the high PAPR problem into a notable advantage, improving system MSE and BER performance. This dual-functionality approach, enabled by the MGCC technique, not only mitigates high PAPR but also enhances channel estimation accuracy, making it a low-complexity solution suitable for next-generation wireless devices. Because the selection of peak-powered subcarriers is determined entirely by the transmitted signal’s characteristics, the approach eliminates the need for receiver-to-transmitter feedback, thereby simplifying the overall system design. Performance evaluations under Rayleigh and Rician fading channels reveal that the proposed scheme performs robustly across diverse conditions. Simulation results demonstrate that optimizing companding parameters for Rayleigh and Rician fading channels effectively reduces PAPR, improves error performance, and ensures accurate channel estimation. Although system performance degrades at low SNR with fewer channel taps, the Rician fading channel exhibits slightly better performance compared to the Rayleigh fading channel. Overall, the proposed DACE scheme enhances system performance from low to high SNR, provided an appropriate number of channel taps is used for the given OFDM subcarriers. Our findings further highlight the adverse impact of receive antenna correlation on system performance. The results show that while low correlation can be tolerated, higher correlation leads to substantial BER degradation, ultimately nullifying the diversity advantage in extreme cases. The integration of PAPR reduction and channel estimation into a single, computationally efficient framework represents a significant advancement in wireless communication systems, offering a practical and innovative solution for modern low-complexity devices.

## Data Availability

The data that support the findings of this study are available from the corresponding author upon reasonable request.
